# The Role of Scientific Expertise in COVID-19 Policy-making: Evidence from Four European Countries

**DOI:** 10.1007/s11115-022-00614-z

**Published:** 2022-04-11

**Authors:** Ron Hodges, Eugenio Caperchione, Jan van Helden, Christoph Reichard, Daniela Sorrentino

**Affiliations:** 1 Birmingham Business School, University of Birmingham, University House, Edgbaston Park Road , Birmingham, B15 2TY UK; 2 Department of Economics ‘Marco Biagi’, University of Modena and Reggio Emilia, Via Jacopo Berengario 51, I-41121 Modena, MO, Italy; 3 Faculty of Economics and Business, University of Groningen, Nettelbosje 2, 9747 AE Groningen, Netherlands; 4 University of Potsdam, August-Bebel Str. 89, 14482 Potsdam, Germany; 5 Department of Business and Law, University of Siena, Piazza S. Francesco 7-8, 53100 Siena, Italy

**Keywords:** COVID-19 policy making, Scientific advice, Political logics, Scientific logics

## Abstract

Immense uncertainty and the need for drastic interventions cause politicians to rely heavily on scientific advice for underpinning or legitimating their COVID-19 decision-making. This paper explores the role of scientific advice in this policy field in Germany, Italy, the Netherlands and the UK. It shows that scientific advice is based on the disciplinary, mainly medical, backgrounds of advisors but is also influenced by social and economic values, which are core to what politicians find important. During the pandemic a growing gap between scientific advice and political decisions is observed.

## Introduction

This paper focuses on the role of scientific expertise in formulating COVID-19 policies. It provides an understanding of how different logics and rationalities of scientists and policy-makers may be combined or give rise to tensions in situations of crisis^1^. An international perspective is provided by comparing experiences in Germany, Italy, the Netherlands, and the United Kingdom, intending to shed light on similarities and differences to help deepen our understanding of policymaking in times of complexity and turbulence (Ansell et al., 2021).

Our analysis includes (a) a review of institutional patterns of collaboration of academic bodies and their contribution towards government policymaking; (b) consideration of the tensions between political and scientific logics in the formulation of government policy; and (c) evidence of the politicization of scientific institutions, including the presence of academics in the media and their apparent impact on policymaking. We study the role of scientific advice in policymaking with regard to measures against the spread and impact of infections. Policy measures for economic support are disregarded. Our paper builds on research that examines COVID-19 policymaking from an international comparative perspective, such as the recent special issues of *International Review of Administrative Sciences* (editorial by Kuhlmann et al., 2021) and *International Journal of Public Administration* (editorial by Edwards and Ott, 2021). Our paper has a more specific focus: rather than the backgrounds, content and outcomes of COVID-19 policymaking, it concentrates on the role of scientific advice in this policymaking.

We contribute to knowledge by providing a comparative assessment of the links between academic expertise and government policy in four European countries which have all been hit by the health, social and economic consequences of COVID-19. We do not seek to suggest that one country or another adopted a ‘better’ approach to combating the virus. However, our observations can form the basis of more detailed comparative international studies on specific policies applied by governments during the crisis.

Our study relies on a qualitative methodology based upon an analysis of governmental documents and those of health authorities, scientific bodies and universities, supplemented by media reports. Rather than developing hypotheses about possible causal links between the theme of our study and its potential antecedents, as in positivist research, we suggest theoretical lenses for our investigations, and iterate between these lenses and the empirical findings for deepening our understanding of the phenomenon at hand. This reveals an interpretive approach (see Ahrens and Chapman, 2006) for studying the role of scientific advice in COVID-19 policymaking. Our theoretical lenses will be introduced and justified in the next section. The selection of countries for our study aims to cover a broad spectrum of administrative traditions and cultures (Kuhlmann et al., 2021, pp. 504–506): the UK with an Anglo-Saxon tradition of majority regimes and quite centralized government functions; Germany with a highly legalistic federal continental system and with a strong position of states (Länder); Italy with a Napoleonic continental system of rule-based governance; and the Netherlands with a Nordic system that emphasises consensus among governmental actors.

Our study aims to address two sets of research questions. First, what are the tensions between political and scientific logics in COVID-19 policymaking, and how do these logics interact? Second, has the relationship between these logics changed during the pandemic? If so, for what reasons and how has this influenced COVID-19 policymaking?

The next section suggests theoretical lenses about the role of scientific expertise in the formulation of public policy, particularly in situations of crisis. The third section provides case studies of COVID-19 policy measures adopted and the role of academic expertise in the formation of policy in each of the four selected countries. The fourth section presents our analysis and discussion. The final section briefly concludes our paper.

## The role of scientific expertise

Policy-makers need to base their decisions about urgent health challenges on scientific knowledge, primarily on medical expertise but also on knowhow from other scientific fields such as economics, sociology or law. Policy-makers therefore may rely on advice from scientific advisors or experts. The provision and usage of such expertise are, however, not without tensions and challenges.

The use of scientific expertise in government policymaking is discussed in a variety of interlinked ways in the literature, often highlighting the conflicting attributes of the policy and scientific communities. Looking at scientists’ and policy-makers’ rationalities in terms of logics of reasoning allows us to focus on how these logics interact in situations of crisis, how scientific logics are embedded in political decision-making and the tensions between them when ‘*Speaking Truth to Power*’ (Wildavsky, 1979). Politicians seek policies that avoid blame or demonstrate their value to citizens (Flinders, 2021). Experts seek an appropriate response based upon professional standards in medical, public health and crisis decision-making, which may be influenced by responses to earlier epidemics.

Policymaking is driven strongly by values, seeking to maintain power and control and to support the respective political interests. Politicians follow their particular political logics, seeking to develop a shared sense of support and belief, often through simple, unambiguous messaging. In contrast, science thrives on disagreement and development, which emphasises knowledge based upon facts and through the testing of ideas against empirical evidence, acknowledging complexity and contingencies (Albaek, 1995; Bogner & Menz, 2021).

Van Dooren & Noordegraaf (2020) question the ability of science to provide a supporting role in policymaking during the COVID-19 crisis. They posit that science in normal times is ‘slow, contentious, collective, and sensitive to complexity’, while crisis science needs to be ‘fast, univocal, personalized and direct’ (p. 610). They argue that scientific impact needs to be actively staged during a period of crisis.

This staging of science notion suggests that the interaction between scientists’ and policy-makers’ logics may develop differently during the various phases of a crisis, with different degrees of reliance on scientific knowledge in formulating policies. In the early stage of a crisis, politicians may rely strongly on scientific experts because they feel a need to react quickly with policy measures and often they cannot draw directly on available common knowledge. This leads government policymaking to embed scientific logics significantly, often by relying upon the support of a few leading scientists.

Later, scientific responses to politicians’ requests become more diversified and scientific experts offer diverging recommendations. Academic advisors often do not speak with one voice, particularly in situations of complexity and uncertainty. Researchers come to deviating observations and findings; they emphasise different aspects of a complex situation as they shed light on diverging issues due to their particular expertise. In any event, empirical research findings are not always unequivocal. Politicians therefore have the opportunity to ‘shop around’ in the scientific market place. Zaki & Wayenberg (2021) present an analysis of ‘epistemic venue-shopping’ to the process of identification, selection and use of scientific expertise in policy formulation. Furthermore, the ambition of some academics may influence them to seek media attention. When advice about issues is contested among academic experts, such as about wearing face masks or the closure of schools and shops, this gives room for political interests to selectively find support for pre-determined policy choices. Furthermore, if the general public believe that ‘experts disagree’ this might be transformed to a view that ‘experts do not know’ or that ‘experts are usually wrong’. This leaves open the potential of reliance on faith, tradition or propaganda to determine ‘what we know to be right’ about COVID-19 (Lee, 2020). All this considered, the desire of governments to provide a univocal and unequivocal message may lead to the exclusion of those scientists who disagree with the dominant perspective. “Not every scientist is allowed on stage” (Van Dooren & Noordegraaf, 2020, p. 612).

The influence of scientific advice on policymaking also depends on structural and political patterns of government and administration. The relevance of science to government decisions may be higher in coalition regimes and in more pragmatic and issue-oriented governments than in one-party governments with populist attitudes.

Distance and differences between scientists and politicians are not always the same. Some scientific bodies (e.g. those under control of government) are closer to policymaking, and may be less likely to directly challenge or contradict existing policies than those researchers who are more distanced from government funding and influence (e.g. Cairney, 2021). Thus, we might expect that political logics will dominate in later stages of a crisis, as political interests will play an important role in the choice of those scientists that governments use to inform and report on policy decisions. Politicians gain more choice options and become more autonomous from scientific consultations, selecting the politically most appropriate measures.

Moreover, as scientists’ and politicians’ logics interact, the boundaries between academic advice and political decision-making may become blurred. This might occur because influential advisors enter the political arena when they cannot resist looking at broader sets of issues than those within their expertise. The complexity of the crisis is emphasised by models and measurements drawn from disciplines beyond epidemiology and public health. Statistical data and predictions from economic, educational and psychological sources redirect public attention away from medical issues. Scientific advisors are therefore caught in more intensive competition to influence policy decisions (Van Dooren & Noordegraaf, 2020).

## Country Case Studies

### COVID-19 figures and public policymaking

The four countries in this study have suffered similar experiences to each other and to many other countries around the world. Table 1 shows cases, deaths, vaccinations and hospitalization rates in each country. The figures provide only a broad indication of impact as differences in data definitions and testing regimes reduce comparability between countries.

**Table 1:** COVID-19 Cases, Deaths, Vaccination & Hospitalization Data

	GERMANY	﻿ITALY	NETHERLANDS	UNITED KINGDOM
Cumulative COVID-19 cases and deaths				
Cases per million inhabitants	162,900	206,568	354,746	273,491
Deaths per million inhabitants	1,445	2,534	1,256	2,356
Daily Case Rates per million inhabitants				
Case rate peak	2,435	3,005	7,298	2,681
Date of case rate peak	14 Feb 22	16 Jan 22	12 Feb 22	5 Jan 22
Case rate: 20th Feb 2022	2,065	862	2,896	629
Vaccination ratio: Fully vaccinated	74.5%	78.2%	71.9%	71.7%
Vaccination ratio: booster	55.8%	61.1%	51.8%	55.8%
Weekly Hospitalization Rates per million inhabitants				
Hospitalization rate peak	156	306	124	432
Date of hospitalization rate peak	24 Dec 20	23 Mar 20	1 Dec 21	13 Jan 21
Hospitalization rate: 20th Feb 2022	74	107	71	127

Source: Our World in Data (GCDL/University of Oxford) https://ourworldindata.org/coronavirus-data

Updated to 20th February 2022 or nearest available date

Daily Case Rates represents 7-day averages of confirmed cases

Vaccination ratios are based upon percentages of total populations

Fully vaccinated rates are based upon the vaccination protocols in each country

Booster vaccination rates are based upon doses in addition to the full vaccination protocols

Weekly Hospitalization Rates are based upon new hospital admissions in the previous week

Table 1 shows that cumulative per capita COVID-19 cases are relatively high for the Netherlands and the UK, they are low for Germany, with Italy in an intermediate position. Cumulative per-capita COVID-19 deaths are relatively high for Italy and the UK and low for Germany and the Netherlands. Daily case rates have been at their highest in all four countries in 2022, reflecting the spread of the omicron variant. However, current hospitalization rates are below peak levels set in earlier waves. Italy has the highest percentage of its population fully vaccinated and in terms of its booster vaccination rate.

There are many COVID-19 policy similarities across the four countries. These include lockdown measures, with full or partial closure of educational institutions, parts of the retail sector, hospitality businesses and entertainment venues. All four countries applied restrictions to meeting people both inside and outside their homes. There were similar demands or requests to adhere to social distancing and to wear face masks. All adopted emergency measures to support their healthcare services including additional intensive care facilities, personal protective equipment and, later in the pandemic, nationwide vaccination programmes. Restrictions on travel were imposed together with additional border controls to reduce the risks of importing the virus. The pandemic has developed in all four countries through several waves, as a result of new variants, seasonal effects and the adoption or release of restrictions.

Despite these similarities of COVID-19 policymaking, there are also striking differences. Given the scope of our paper, we can only outline some of them. Constraints related to staying at home during a lockdown were, in general, more severe in Italy than Germany, with the UK and the Netherlands in an intermediate position. The UK and the Netherlands were reluctant to apply severe measures for constraints in early March 2020, due to their belief in realizing ‘herd immunity’, while the policy reactions in Germany and Italy were relatively less hesitant (Source: Dutch TV news show ‘Nieuwsuur’, 28 & 29 October 2021). Herd immunity anticipates that the majority of citizens may be infected without becoming seriously ill, resulting in immunity for large parts of the population. The approach was abandoned in both the UK and the Netherlands before the end of March 2020 (Farrar, 2021, pp. 94–96; Pattyn et al., 2021 pp. 596, 603). Countries also differ in the extent of centralisation or decentralisation of their governmental systems in responding to the COVID-19 pandemic. The UK is the most centralized, despite some devolution of powers to Scotland, Wales and Northern Ireland; Germany the most decentralized, with significant powers given to its state governments; with Italy and the Netherlands in intermediate positions.

### Germany

#### Institutional background

Compared to the other countries, Germany’s hospital capacities were in a less critical situation. However, the complex German federal system turned out partly to hinder fast and resolute reactions to fight the pandemic. The Federal Government does not have any direct competences to handle the pandemic (Kropp & Schnabel, 2021). It can only set the legal framework (the Federal Infection Act) and co-finance policies against the pandemic. The 16 state governments, together with local governments, are responsible for the execution of public tasks like health care. In the case of COVID-19, the state governments implemented the various policies rather independently and not with consistent rigour. They were only loosely coordinated in the regular Prime Ministers’ conferences and softly guided by the Federal Government. As a result, measures to limit the COVID-19 infections differed between the states and caused irritation and sometimes discontent in the population.

Another cause of ineffective handling of the pandemic is the rather inflexible, bureaucratic and slow administration at state and local levels. Local health authorities were understaffed and lacked adequate digital tools, so they were unable to track and follow-up the contacts of infected people. Due to the highly decentralized testing, tracking and recording of infections, officially registered infection rates were sometimes much lower than the real ones. In addition, school administrations were unable to equip schools and teachers with laptops and software or to provide all class rooms with air filters. Legal restrictions sometimes hampered effective COVID-19 fighting, such as the limited usability of the ‘Corona App’ as result of excessive data protection legislation.

#### The role of scientific expertise

The major institution with a scientific focus in Germany in the area of infectious pandemics is the *Robert-Koch-Institut* (RKI), a higher federal authority under direct control of the federal Ministry of Health with over 1,000 employees, among them almost 500 scientists. It provides daily epidemiological data about infection rates etc. Its influence on government decisions is high, as it is the major supplier of medical data and of policy recommendations. As a federal agency, on the other hand, the RKI is dependent on funding, staffing and strategic orders from Federal Government and has therefore only limited autonomy (Dostal, 2020). More recently, the new Federal Government of 2021 established a particular task force, consisting of 19 experts, mainly from virology and other medical disciplines but also from pedagogics and health care authorities.

There are also some semi-autonomous expert panels providing science-based recommendations to the government (e.g., on ethical or vaccination issues). Additionally, some researchers related to virological and epidemiological institutions are quite influential on policymaking. Advice is not always univocal, giving politicians room for ‘shopping’ around. Prominent scientists also appear regularly ‘on stage’ at talk shows and discussions on TV, radio and in the newspapers. A few politicians with an academic background in medicine, who are members of the German *Bundestag*, played a special role in the interplay between science and politics. Quite regularly, they commented on government decisions and gave recommendations based upon their scientific expertise and influenced by their party-political background. On top of this, the current federal minister of health has a background in virology.

In the first wave of the pandemic, the decision mode of Parliament and Government was predominantly an administrative one (procurement of masks or lockdown measures). Political conflicts were consequently less relevant (Bogner & Menz, 2021, p.123). Later on, politicians had to cope with more complex and value-laden problems. Accordingly, the advice given by scientific experts was more varied and sometimes even contradictory (e.g. school closures versus maintaining education). Consequently, politicians were confronted with diverging advice giving room for autonomous political decision-making. Thus, the legitimating impact of science on political decisions was particularly large in the early phases of high turbulence and problem novelty. Later, this legitimating impact decreased, as the voices of science became more pluralistic and the political system returned to its usual ‘political mode’ with its value-based controversies and fights. Consequently, the divergence between scientific and political logics increased during the course of the pandemic.

After re-election of the federal parliament in autumn 2021, COVID-19-related policymaking shifted into a rather slow and passive mode, before the new government was finally established. In this power vacuum between old and new government, the influence of scientific advisors was particularly weak, because the previous government was very reluctant to take rigorous decisions. As a result, infection rates increased significantly and hospital beds became scarce. The new government tends to prefer a coordinated COVID-19 policy style and to be more open to scientific advice.

### Italy

#### Institutional background

The Italian health care system has become increasingly decentralised and inhabited by many private actors during the last two decades. One of the most urgent challenges posed by the pandemic has been to align, and find a consensus for, central and local governments’ reaction to the health emergency (Sancino et al., 2021). Political confusion emerged as the interactions between governmental levels on some sensitive issues (e.g., imposition of lockdowns) were unclear at the beginning. Longstanding regional differences on healthcare capacity became worrying, as even Northern regions, supposedly managing a wealthier and technologically advanced healthcare system, were caught unprepared (Rubinelli, 2020).

A centralization of authorities was enacted, whereby the Italian Government could issue legislative provisions through a leaner procedure that eventually oversteps parliamentary approval. However, regions (and to some extent municipalities) were gradually involved in making decisions on issues such as the closing of schools, local transport, freedom of movement, and economic support for companies (Palermo, 2021). Moreover, regions are entrusted with managing fundamental operational activities (e.g., contact tracing, lockdown supervision and the organization of vaccination hubs).

The health and socio-economic issues arising during the pandemic have exacerbated not only the regional differences on the healthcare, economic and cultural levels, but also the instability of the central government (Di Mascio et al., 2020). Italy experienced a governmental crisis during the second wave, which led to the appointment of a new Prime Minister, Mario Draghi, former President of the European Central Bank.

#### The role of scientific expertise

For the purpose of research, control and technical-scientific consultancy in the field of public health, the Italian Government can rely on the *Istituto Superiore di Sanità* (ISS), an independent centre where researchers, technicians and civil servants work. The ISS, headed and directed by scientific experts, has played a key role in orienting public policies during the pandemic. The Italian Government has also established a technical-scientific committee, TSC (*Comitato tecnico-scientifico*) on 5th February 2020, entrusted with consulting and supporting activities to overcome the emergency. The TSC is chaired by the President of the Higher Health Council of the Ministry of Health, and composed of twelve scientific experts, five of whom are affiliated with governmental bodies. The TSC is constantly consulted by the Government on sensitive issues (e.g. the vaccination campaign), and it defines a set of indicators to allow the Government to evaluate, on a weekly basis, the conditions of each region and the opportunity either to impose tougher restrictions or to ease them. Government decision-making has largely relied on the TSC recommendations, explicitly referring to them in public announcements. This occurred especially during the first two waves, when harsh measures were taken, while the focus on the TSC recommendations was nuanced in favour of more emphasis on economic and social issues by the third wave. Although the ISS and the TSC are deemed to cooperate, this latter plays a key role in orienting policymaking.

Additionally, several other task forces of independent scientists with interdisciplinary expertise have been set up at key Ministries.

Moreover, scientists are regularly hosted in Italian talk shows and frequently resort to social media to publicize their opinions on most debated topics. As the areas of expertise of these leading scientists are represented on the TSC, public debates have often relied not only on discussions between experts and non-experts, but also between experts who are officially engaged in the management of the pandemic and other experts. This created a knowledge contrast between the individual experts’ opinion and the TSC pronouncements on the most sensitive issues. The apparent lack of conflict between governments and scientists involved in policymaking suggests that the former had the opportunity to ‘shop’ around.

Knowledge contrast has sometimes resulted in a confusing picture for the public, creating distrust of the governmental management of the pandemic and a perception that the TSC is an “appendix” of the Government. Non-experts, who (nevertheless) boast scientific knowledge, also provided contrasting opinions, thus exacerbating this confusion. Furthermore, scientists’ interventions have not always concerned matters strictly related to their areas of expertise. For example, virologists and epidemiologists have commented on the planned timing of the vaccination campaign. Grounded on personal beliefs, such debates run the risk of making scientists’ interventions ideological, and creating value-based conflicts between academics.

### Netherlands

#### Institutional background

Dutch municipalities and provinces have substantial autonomy, but within boundaries set by central government. Its political system is consensual, characterized by intensive consultations between political parties and governmental layers. This is reflected in the governmental approach to the pandemic: regionalisation supplemented with central coordination and control. The Dutch health care system went through serious cost reduction measures since the mid-2000s, which decreased the number of hospital beds and intensive care units, also in comparison with other countries (Pattyn et al., 2021, pp. 599–600). During the first lockdown in March-April 2020, intensive care capacities were extended, but nation-wide coordination was needed to avoid regional shortages. The health care system became impoverished throughout the pandemic and induced the postponement of non-COVID-19 treatments, and shortages in personnel capacities due to absenteeism and departure of healthcare staff.

#### The role of academic expertise

Prime Minister Rutte stipulated the importance of medical expertise in COVID-19 policymaking: “The answer to all questions starts with the knowledge and experience of experts.” (Source: press release 16 March 2020). The Dutch Institute for Public Health and the Environment (RIVM) provides figures about the COVID-19 pandemic, including the R-value, the number of infections and the death rate, on a weekly, sometimes even daily basis, and presents predictions about pandemic-relevant indicators (Source: RIVM.nl).

In the Netherlands, a specific medical task force, called the Outbreak Management Team (OMT) was created at the start of the pandemic for providing publicly available advice, and this group of about forty medical specialists became powerful. Power is, however, not riskless. OMT members were forced by their chairman, RIVM director Professor Jaap van Dissel, to speak with one voice. So, there was little room for individual members to show minority viewpoints, which conflicts with their scientific logic. Another complication was that the OMT chair regularly entered the political arena, due to his close contacts with ministers, but also because these politicians were putting pressure on him to defend or not offend certain ideas about COVID-19 measures as instigated by politicians, such as the re-opening of primary schools (Volkskrant, 30 January 2021). These examples reveal tensions between scientific and political logics.

The boundaries between politics and medical expertise became contested, as some top medical specialists often appeared in the media to give their opinion, not only on medical problems but also on non-medical issues, such as the need to open schools due to the educational and social values attached to face-to-face education. So, experts in specific disciplines were becoming public intellectuals in a more general sense. In addition, at the beginning of the pandemic, OMT advisors explicitly anticipated on issues of support among citizens about restrictive constraints, and in this way they entered the political domain (source: TV news show ’Nieuwsuur”, 29 October 2021). This relates to the staging of academic knowledge as indicated in the second section. In the Netherlands, four medical experts, including the OMT chairman, were clearly at the top of this scientific pyramid. One of these scientists, Professor Ernst Kuipers, was even appointed as the new minister of public health in January 2022, which can be seen as the ultimate form of scientific staging.

Next to OMT, a permanent advisory body for the government on public health, the Health Council of the Netherlands (de Gezondheidsraad, http:/www.gezondheidsraad.nl) has specific tasks related to the pandemic, such as giving advice on vaccination sequences. There is also competition about COVID-19 expertise from other scientists by the so-called Red Team (Source: https://www.c19redteam.nl/). In January 2021, the Red Team advised that the older and more vulnerable should be separated from relatively younger and healthier people, in order to protect the former and give space for living to the latter (Herstel.nl). This initiative was heavily criticized due to its ethical issues and its lack of feasibility, and thereafter, the Red Team became almost invisible in the media.

The role of scientific advice, especially from the OMT, changed in the course of time. At the start of the pandemic, advice about lockdown constraints was used by political leaders to justify policymaking, in which health care values were core. However, social and economic values became more important later in the pandemic and this induced a gap between scientific advice, characterized by caution, and political decision-making in which giving more freedom to citizens and businesses became increasingly important.

### United Kingdom

#### Institutional background

The UK is one of Europe’s most centralised democracies, although matters of public health are delegated to the devolved administrations of Scotland, Wales and Northern Ireland. This results in variations of policy across the UK, although all follow a general pattern of multiple lockdowns and partial release of restrictions. The UK Government has a Civil Contingencies Committee (known as COBR or COBRA) to handle large scale emergencies, which is normally chaired by a government minister (www.instituteforgovernment.org.uk/explainers/cobr-cobra).

The UK was ill-prepared for any severe medical crisis. The UK Government had slashed public expenditure following the financial crisis of 2008. There was no available test-and-trace system and stocks of personal protective equipment were at minimal levels. Local governments suffered particularly large financial cuts and local public health experts were insufficiently well involved in the design and early operational use of the test-and-trace system (HSCC-STC, 2021, p. 76).

#### The role of scientific expertise

Government mantra, particularly in the early stages of the pandemic, has been that they are ‘following the science’. The Scientific Advisory Group for Emergencies (SAGE) is a sub-committee of COBR and has taken a leading role in providing scientific advice during the pandemic. It is chaired by Professor Sir Patrick Vallance, the UK Government’s Chief Scientific Advisor and its vice-chair is Professor Sir Chris Whitty, the Chief Medical Officer for England. Vallance and Whitty, as part of a small group of senior SAGE members, became the public face of the scientific community during the pandemic. They have often appeared with the Prime Minister at televised presentations from Downing Street, providing scientific data in support of the UK Government’s policies, and in TV and radio news and science programmes.

SAGE has 115 participants listed on the government’s web-site (www.gov.uk/government/organisations/scientific-advisory-group-for-emergencies). Of these, 45 are members of academic institutions and another 27 have professorial titles, working mainly in advisory capacities for government and public sector bodies. Dominated initially by modellers and epidemiologists, its membership and its advisory sub-groups widened during the pandemic. Advisory bodies reporting to SAGE include groups dealing with infectious disease modelling, virus threat risk assessment & mitigation, immunisation strategies & priorities, and behavioural issues.

Other non-government linked bodies of scientists, medics and academics have commented on government policies during the pandemic. These include the Royal Society (https://royalsociety.org) which has published many academic papers, and the self-named Independent SAGE which has issued reports and press releases, often critical of government policy (https://www.independentsage.org/). Ad-hoc groups of academics, scientists and medics have issued joint statements, including criticism of the early removal of restrictions by government. Scientists commenting, from their personal perspective, on government policies have become a regular feature of news events in the media, blurring the boundary between scientific and political logics.

In summary, SAGE represents the most influential scientific advisory body to the UK Government on COVID-19 matters, with its senior members as core advisors. Others appear to have a more peripheral role, in some cases providing an academic forum to include the views of core advisors, with others taking a more openly hostile view of government policy (Cairney, 2021).

The UK Government was criticised extensively for the delay to its first national lockdown. SAGE has not been immune from this criticism. It has been suggested that SAGE supported a ‘slow and gradualist’ approach to the imposition of restrictions, and that Vallance effectively supported a herd immunity approach during a press conference on 12 March 2020 (HSCC-STC, 2021, pp. 32–33). However, a member of SAGE, referring to its meeting two days previously, stated that the delay and mitigation elements of the Government’s plan ‘looked dangerously inadequate’ (Farrar, 2021, p. 104).

Differences between scientific advice and policy decisions began to emerge later in the pandemic. On 21st September 2020, SAGE advised that a 2 week ‘circuit-breaker’ lockdown be imposed because cases and hospitalisation numbers had risen after the summer. The UK Government ignored this advice (HSCC-STC, 2021, p. 49), delaying lockdown until November. In October 2021, there were calls from health-care providers, the British Medical Association and Independent SAGE for the reintroduction of compulsory restrictions, such as mask-wearing and social distancing. Restrictions were reintroduced in England only in mid-December. Wales, Scotland and Northern Ireland have generally imposed restrictions earlier and released them later than England. Restrictions have begun to be removed from January 2022, emphasising the UK‘s reliance on its booster vaccination programme to counteract the omicron variant. SAGE has not directly contradicted these policies, but warns of uncertainties in the trends of new infections and hospital admissions and of the social and behavioural impacts of lifting restrictions (SAGE, 2022).

## Comparative analysis and discussion

The theoretical lenses in the second section and the four country studies in the third section provide the basis of our comparative analysis and discussion of findings. An overview of the individual country findings is shown in Table 2.

**Table 2:** Overview of Country Findings

	Germany	Italy	Netherlands	UK
Scientific institutions and professionals	RKI with major impact and limited autonomy from government. Other expert groups with increasing impact during pandemic. The new government established a more autonomous advisory body.	TSC as a special task force and ISS as a permanent advisory body, both close to government. Various autonomous expert groups.	OMT as a special taskforce with RIVM and health council as permanent advisory bodies; all close to government. Also autonomous, interdisciplinary expert groups.	SAGE, as part of COBR, close to government. Also government advisory groups and non-governmental bodies, the latter remote from government.
Logics of scientists and politicians	Generally conflicting logics. Scientists in government agencies follow more political logics.	Generally cooperating, especially with those officially involved in policymaking.	Hints about tensions between independence of experts and engagement in policymaking.	Implicitly disclosed, general assertions by politicians and facts and figures from scientists.
Staging of scientists	Staging of scientists was apparent.	Staging of scientists was apparent, also beyond expert domains.	Staging of scientists was apparent, also beyond expert domains.	Staging of scientists was apparent.
Dynamics of scientific advice during the crisis	The 1st wave saw univocal political decisions, with legitimation from scientific experts. More value laden issues and conflicting advice later.	The 1st and 2nd waves saw strong reliance on TSC advice. Later, policymaking gave more space to economic and social values.	Scientific advice for justifying policymaking in the 1st wave. Gaps between advice and political decision-making due to conflicting values later.	Ambiguous information use of scientific advice at the beginning of the pandemic. Later, scientists were more cautious than politicians.
Boundaries between scientific experts and politicians	Increasingly blurred boundaries and diverging scientific advice.	Scientists have debated aspects beyond their domain.	Blurring boundaries because scientists anticipate on support among citizens.	Individual scientists commenting in a personal capacity blur the boundaries

The COVID-19 pandemic affected all four countries in a similar way, although relative numbers of infections and deaths diverged to some extent (see Table 1). One reason for divergence was the starting time of the pandemic. While Italy and UK were confronted very early with the outbreak, Germany had more time to get prepared. The patterns of health-related policymaking differed in the four countries. While policies in Germany were formulated and implemented in a decentralized way, the UK and the Netherlands followed a more centralized approach. Italy adopted a relatively strict centralization of policymaking, though regions were increasingly involved in policymaking and entrusted with managing fundamental activities. The extent of regionalisation of policymaking was also diverging. Central steering and coordination were dominant in the UK, Netherlands, and Italy during the pandemic, while Germany was characterised by a more decentralised approach (see also Kuhlmann et al., 2021, for a comparative perspective on COVID-19 policymaking associated with governmental traditions and cultures).

Scientific advice for policymaking was prominent in all four countries. While Italy and the Netherlands created specific scientific task forces for giving advice, in the UK and in Germany existing institutions provided advisory support. In all four countries these advisory bodies were closely connected to governments, due to their funding and organizational position, although they seemed to be less autonomous from government in Germany and Italy than in the Netherlands and the UK. Advice from such experts’ panels has turned out to be an important precondition for effective COVID-policies, as politicians can less easily deviate from such authoritative advice (Toeller et al., 2021).

In addition to these primary advisory bodies, other advisory institutions and groups were active, with a varying extent of autonomy towards their governments. The virus outbreak and spread became a top item in the media, ranging from newspapers and television and radio shows to social media. These media were actively staging scientific experts for enlightening virological and epidemiological backgrounds and for providing reflections and recommendations on governmental policymaking. A few experts in each of the countries became media personalities, as they personalized the scientific information and in this way contributed to its dissemination (Van Dooren & Noordegraaf, 2020). Due to their expertise, many of them were probably more trusted by the public than leading politicians, but their predictions and opinions were disputed and disapproved by those citizens who felt disadvantaged by the constraints put on them through government policies.

The logics underlying actions for fighting the virus outbreak and its spread differ between scientific experts and policy makers. The scientific advisors in the UK seem to emphasize that ‘facts and figures’ matter in interpreting infection patterns and possible impacts of interventions, whilst leading politicians, particularly in later stages of the pandemic, downplay the possible health impacts to give comfort to their potential voters. In Germany, Italy and the Netherlands, we observed that scientists on the one hand were adhering to claims they found defendable from their disciplinary background but on the other hand, especially when their formal position was connected to political decision-making, were inclined to engage themselves with political considerations about proposed measures. So, political logics seem to be sedimented upon rather than integrated with their original scientific logics. Advisors from the Dutch OMT, for instance, explicitly anticipated a lack of support amongst citizens about restrictive constraints at the beginning of the pandemic, when they would have preferred relatively more severe interventions, and in this way they entered the political domain. Experts appointed by the Italian Government rarely released opinions conflicting with government decisions. In a more general sense, we see that advisors followed not only their particular logics as scientists, but were also receptive to the broader politically-centred logics of their clients, such that the boundaries between scientific advice and political decision-making became blurred. Less common are examples of politicians being able to combine the political and scientific logics, although a few members of the German Bundestag operated as intermediaries between science and politics due to their medical backgrounds.

In accordance with our expectations presented in the second section, politicians did rely strongly on scientific experts in early stages of the pandemic, while later on scientific responses to politicians’ requests became more diversified and some experts also distanced themselves from political decision-making. In all four countries, politicians needed scientific advice for legitimating measures that heavily constrained the lives of citizens and businesses in the beginning of the pandemic. The need to protect health and save lives, coupled with substantial uncertainty about the effectiveness of potential measures, were conducive to gaining citizen support for interventions. At later stages the willingness of citizens to follow government provisions and the recommendations of experts declined remarkably, particularly in Germany, Italy and the Netherlands where citizens tried to escape from the rules and became sceptical against COVID-19 measures, e.g. with regard to vaccination. Furthermore, health values increasingly had to compete with social and economic values, giving rise to more diverse advice from academic experts. Consequently, the gap between politicians and experts expanded. While the former became focused on serving economic and social values, the latter, especially those coming from medical disciplines, claimed to keep health values at the centre.

Findings from this comparative analysis lead us to challenge the traditional view on the role of expert advice in decision-making as grounded solely on ideas of scientific rationality. According to this view, decision-makers know what they want to achieve – their goals are clear – and they are interested in the actions that potentially help to achieve these goals (e.g., Burchell et al., 1980). Experts can support decision-makers by identifying these ‘effective’ actions. However, in circumstances of large uncertainty about the effectiveness of actions, and the contestability of goals, this rational view on the role of expert advice in decision-making is challenged. Put differently, crisis management might require another view.

The COVID-19 pandemic was largely unprecedented, and there was limited knowledge about measures to constrain the pandemic (Ansell et al., 2021). Various issues were at stake, such as: whether to protect only the most vulnerable citizens and let the virus run its course to achieve herd immunity; how to prevent young people from becoming infected and passing infection onto more vulnerable people; the effectiveness and feasibility of testing and tracing systems for early discovery of infections; and the distancing rules necessary for sports and cultural events to mitigate virus outbreaks.

Additionally, governments were struggling with the diverging weights attached to public health on the one hand and economic and social values on the other hand. At the beginning of the pandemic there was a strong sense of urgency among people for accepting drastic measures for fighting the pandemic, so public health was core, but at later stages economic and social values became more important. An increasing sense of ‘covid fatigue’ appeared to develop as citizens became weary of restrictions on movement and activities. This is illustrated in demonstrations against the reintroduction of COVID-19 measures. The Italian and UK Governments placed particular reliance upon the effectiveness of their vaccination programmes, including booster doses for citizens, to avoid reintroducing severe restrictions.

The effectiveness of governments in controlling the pandemic seems to be enhanced by a pragmatic decision-making style based upon collaboration between political authorities and professional expert bodies, together with successful sense-making and communication with the public (Christensen & Lægreid, 2020a, b). Politicians must present plausible narratives, convincing arguments and communicate with the public in an unambiguous and action-related manner (see Weick et al., 2005). Sense-making challenges politicians because they need to convince the public about the appropriateness of proposed actions, but they also have to encourage their citizens to behave in accordance with guidelines, such as working at home, social distancing, testing and vaccination. It also has implications for the role of experts in advising politicians. Suggesting all kinds of contingencies for the effectiveness of various actions is often seen as problematic and may be ineffective in convincing politicians and citizens to take actions that are perceived by scientists to be appropriate.

Based on these reflections, the implications of our comparative findings can be expanded. First, how does the staging of academic experts evolve in circumstances of high uncertainty and contestable goals as in the COVID-19 pandemic? In all four countries, academic experts are active in the media by presenting scientific insights and recommendations on COVID-19 issues. Their appearances, however, diverge depending on the distance they have from political decision-making (Cairney, 2021). Experts who are part of formal advisory boards in their country show reluctance in providing narratives that contradict explicitly those presented by political officials. In contrast, academic experts with a more autonomous position towards government are more likely to criticize official policymaking and to provide divergent recommendations.

Second, how has the role of scientific advice changed in the course of the pandemic? Here we see two opposing forces. On the one hand, knowledge about the effects of COVID-19 measures was very limited at the beginning of the pandemic. So, politicians were seeking convincing messages for their citizens in the light of potential COVID-19 policies. The concept of herd immunity provides an interesting example. Politicians may need a comforting narrative to support the delay of drastic measures like a lockdown. ‘Herd immunity’ was based on a naïve belief that mild COVID-19 infections would help lessen the impact of the pandemic. The uncertainty about the impacts of the pandemic in early 2020, even amongst medical experts, was obviously so high that politicians tried to offer a narrative with a positive tone.

Later, the impact of ‘covid fatigue’ and the resistance against restrictive government actions became stronger. Politicians became increasingly vulnerable to pressures ‘to get back to normal life’. We see in our country studies that, in these circumstances, scientific advisors were, in many cases, far more critical than politicians in their assessment of the impact of relaxing measures.

Third, in what ways have tensions between political and scientific logics impacted COVID-19 related policymaking? There seems to be an asymmetrical relationship between political and scientific logics. In circumstances leading to the ‘bad news’ of imposing severe restrictions, such as a national lockdown, advisors need to have convincing evidence to support the imposition of draconian measures. They have to convince politicians to take these measures, and, if they succeed, tensions between scientific and political logics will be limited. In these circumstances, politicians need scientific advice to justify and legitimate their policies. However, when there is a potential for ‘good news’ about relaxing COVID-19 restrictions, politicians are inclined to be less cautious than scientific advisors in assessing these relaxations. Politicians may feel that they have much to gain from being seen to be leading their citizens out of existing restrictions. More generally, scientists may be more prudent in their assessments. The particular COVID-19 context confronts them with the need to defend positions that are surrounded by high uncertainty. In these circumstances, politicians and scientific advisors also face the risk of being engaged in a blame game. Governments may accuse academic advisors when measures turn out to be ineffective (Flinders, 2021). However, although advisors may have accepted the political rhetoric that policy is led by science, they should have recognized that the science was not unambiguously knowledgeable. In such a context, we observe not only a growing scientization of politics, but also an increasing politicisation of science (Hoppe, 1999).

## Conclusions

Our research has attempted to deepen our understanding of the role of scientific advice in COVID-19 policymaking. Two major messages come out of our study of Germany, Italy, the Netherlands and the United Kingdom.

First, the role of scientific advice changes in the course of the pandemic. In early stages, immense uncertainties about the effectiveness of potential interventions for fighting the outbreak and spread of the virus induced leading politicians to rely heavily on medical expertise for justifying severe constraints on the lives of citizens. However, later in the pandemic, gaps emerged between scientific advice emphasising caution, while politicians increasingly became inclined to promote a relaxation of restrictions to serve economic and social values. At this stage, the logics of scientists, who attach value to evidence and prudence, diverge from the logics of politicians, who seek to comfort their voters with good news.

Second, our study challenges the traditional view on expert advice for underpinning the best possible actions to take for accomplishing well-defined goals. The pandemic provides a context of conflicting values under substantial uncertainty about options for actions. In this situation, expert advice is directed at providing convincing narratives to politicians for guiding their citizens towards proper behaviour. Expert advice then contributes to sense-making rather than the underpinning of quasi-optimal political decision-making.

## Footnotes

1 This paper uses the labels scientific and academic advice as interchangeable. Academic advice is understood as the advice of experts in disciplines that are seen as relevant for considering COVID-19 related issues.
